# Consensus on Language for Advance Informed Consent in Health Care–Associated Pneumonia Clinical Trials Using a Delphi Process

**DOI:** 10.1001/jamanetworkopen.2020.5435

**Published:** 2020-05-22

**Authors:** Amy Corneli, Sara B. Calvert, John H. Powers, Teresa Swezey, Deborah Collyar, Brian Perry, John J. Farley, Jonas Santiago, Helen K. Donnelly, Carisa De Anda, Katelyn Blanchard, Vance G. Fowler, Thomas L. Holland

**Affiliations:** 1 Clinical Trials Transformation Initiative, Duke University School of Medicine, Durham, North Carolina; 2 Department of Population Health Sciences, Duke University School of Medicine, Durham, North Carolina; 3 Duke Clinical Research Institute, Duke University School of Medicine, Durham, North Carolina; 4 Department of Medicine, George Washington University School of Medicine and Health Sciences, Washington, DC; 5 Patient Advocates in Research, Danville, California; 6 Center for Drug Evaluation and Research Food and Drug Administration, Silver Spring, Maryland; 7 Department of Medicine, Northwestern University Feinberg School of Medicine, Chicago, Illinois; 8 Merck & Co, Inc, Kenilworth, New Jersey; 9 Duke Clinical Research Institute, Duke University School of Medicine, Durham, North Carolina; 10 Department of Medicine, Duke University School of Medicine, Durham, North Carolina

## Abstract

**Importance:**

Information to be included in advance informed consent forms for health care–associated pneumonia treatment trials remains to be determined.

**Objective:**

To identify and determine how to describe information to be included in an advance informed consent form for an early-enrollment noninferiority hospital-acquired and/or ventilator-associated bacterial pneumonia (HABP/VABP) clinical trial.

**Design, Setting, and Participants:**

A Delphi consensus process with stakeholders in HABP/VABP clinical trials was conducted using qualitative semistructured telephone interviews from June to August 2016, followed by 2 online surveys, the first from April to May 2017, and the second from September to October 2017. All stakeholders who participated in the interview were invited to participate in the first survey. Stakeholders who participated in the first survey were invited to participate in the second survey. Stakeholders were patients at risk of pneumonia, caregivers, representatives of institutional review boards, investigators, and study coordinators.

**Main Outcomes and Measures:**

Description and consensus of information to be included in advance informed consent forms for early enrollment in noninferiority HABP/VABP clinical trials.

**Results:**

Suggestions from 52 stakeholders about what key informed consent concepts to include and how to explain them were used to create 3 categories to be included in an advance consent form: (1) reassurances on patient health and treatment, (2) rationale for advance consent and early enrollment, and (3) an explanation of noninferiority. At the end of the Delphi process, at least 80% consensus was reached among the 40 stakeholders who participated in the second online survey on each of the statements to include in the proposed consent text. Throughout the process, however, describing and reaching consensus on statements about noninferiority was more problematic than the other categories.

**Conclusions and Relevance:**

The stakeholders endorsed consent language to be used in combination with a strategy for enrolling patients at highest risk for pneumonia before infection onset. Data-driven consent language may help potential participants make informed decisions about their involvement in clinical research and improve enrollment rates, which are necessary to evaluate new treatments and improve patient care. The proposed consent language may be adapted for other trials using an early enrollment strategy and for noninferiority trials.

## Key Points

**Question:**

What information do key stakeholders want in informed consent forms for noninferiority clinical trials on hospital-acquired and/or ventilator-associated bacterial pneumonia, and how should such information be described?

**Findings:**

In this Delphi study of 52 key stakeholders (patients at risk of pneumonia, caregivers, representatives of institutional review boards, investigators, and study coordinators), consensus was reached on how to describe reassurances on patient health and treatment, a rationale for advance consent and early enrollment, and noninferiority.

**Meaning:**

Results of this study suggest that inclusion of information on these concepts in informed consent forms may help potential participants make informed decisions about participating in noninferiority trials of health care–associated pneumonia treatment.

## Teaser

This study uses a Delphi consensus process to identify information to be included in an advance informed consent form for an early-enrollment, noninferiority clinical trial of treatments for hospital-acquired and/or ventilator-associated bacterial pneumonia.

## Introduction

In response to difficulties in recruiting patients for clinical trials in hospital-acquired and/or ventilator-associated bacterial pneumonia (HABP/VABP),^1,2,3,4,5^ the Clinical Trials Transformation Initiative (CTTI), a public-private partnership cofounded by the US Food and Drug Administration (FDA) and Duke University, Durham, North Carolina, developed a novel early enrollment strategy as part of its portfolio of projects in antibacterial drug development.^6^ With this strategy, patients at risk for hospital-acquired pneumonia consent to monitoring by study staff in advance of developing pneumonia. If these patients subsequently develop HABP/VABP, they are randomly assigned to receive an investigational or comparator antibiotic. Although obtaining advance consent for treatment of potential complications that require immediate care is feasible,^7^ enrolling patients early into clinical trials is uncommon. Knowledge about appropriate information to include in advance consent forms for pneumonia treatment trials remains to be determined, particularly for noninferiority trials.^8^

Previous CTTI research suggested that key stakeholders find an early enrollment strategy using advance consent in pneumonia antibiotic trials to be acceptable.^9^ As a component of that research, CTTI also engaged these stakeholders to identify, describe, and reach consensus on essential concepts to be included in an advance consent form for an early-enrollment, noninferiority HABP/VABP clinical trial. We describe here the development of the proposed text for the advance consent form, together with supporting information.

## Methods

We engaged 5 stakeholder groups in the research: patients at risk for pneumonia, caregivers of patients at risk for pneumonia, investigators, study coordinators, and representatives of institutional review boards (IRBs). Information on stakeholder recruitment and selection has been reported.^9^ We used a Delphi consensus process^10^ to identify information to be included in an advance consent form and how to describe that information. We explained to stakeholders that hospitalized patients who were identified to be at risk for pneumonia would be asked to consent to being monitored by study staff to determine if they develop HABP/VABP, and randomly assigned to receive 1 of 2 antibiotics if they develop HABP/VABP. The antibiotics were imipenem, an FDA-approved antibiotic for lower respiratory tract infection, and meropenem, an antibiotic approved for other serious infections and commonly used off- label for treating pneumonia. This study followed the Standards for Reporting Qualitative Research (SRQR) reporting guideline for qualitative studies.

As the first step in the Delphi process, we conducted qualitative, semistructured telephone interviews. The interviewer provided stakeholders with an overview of the proposed noninferiority trial and early enrollment strategy. The interviewer also described the study antibiotics and explained to patients and caregivers that the study hypothesis was that meropenem may not work as effectively as imipenem in the treatment of pneumonia but may have fewer adverse effects. (eAppendix 1 in the Supplement includes the study overview provided to all stakeholders.) Table 1 lists the initial and follow-up topics explored with each stakeholder group. The initial questions asked stakeholders to describe essential information that should be provided in an advance consent form and how to explain that information. The follow-up probes solicited stakeholders’ suggestions on how to explain certain topics, if not initially mentioned. We included the follow-up topics because (1) we expected the need to describe these areas in the advance consent form and wanted stakeholder feedback on how best to explain them, and (2) research has shown that the purpose of noninferiority trials (one of the probed topics) is typically not described in consent documents.^8^

**Table 1.** Interview Topics Explored by Stakeholder Groups

Stakeholder group	Initial topics	Additional follow-up topics
Patients (n = 18) and caregivers (n = 12)	What essential concepts about the early-enrollment HABP/VABP clinical trial should be explained to patients How those concepts should be explained	What information should be provided to patients to explain^a^ Rationale for early enrollment strategy and enrolling patients before they develop pneumonia How patients would be identified Why the 2 antibiotics would be compared
Investigators (n = 7) and study coordinators (n = 5)	What essential concepts about the early-enrollment HABP/VABP clinical trial should be explained to patients How those concepts should be explained	What information should be provided to patients to explain^a^ Rationale for early enrollment strategy and enrolling patients before they develop pneumonia Noninferiority Benefits of early enrollment strategy Why a patient has been identified as a potential participant for the HABP/VABP trial
IRB representatives (n = 10)	What essential concepts about the early-enrollment HABP/VABP clinical trial should be explained to patients	What information should be provided to patients to explain noninferiority^a^

Abbreviations: HABP/VABP, hospital-acquired and/or ventilator-associated bacterial pneumonia; IRB, institutional review board.

^a^ Asked if the topic was not mentioned in stakeholders’ initial responses.

We analyzed the qualitative data using applied thematic analysis.^11^ We first identified the most common essential concepts that stakeholders mentioned and how to describe them before the interviewer probing. Next, we thematically combined those responses with the probed responses on how to explain essential concepts to identify the most common suggestions for describing the concepts. We grouped these suggestions into larger consent categories, focusing on categories that go beyond the Common Rule^12^ elements of informed consent (eg, risks) and that the study team believed should be explained in an advance consent form (ie, noninferiority trials). The study team then drafted consent form text for these categories, taking into consideration stakeholders’ suggestions for explaining essential information.

We then conducted 2 rounds of online surveys with open-ended and closed-ended questions to assess the importance of the consent text and to reach consensus on the advance consent language. All stakeholders who participated in the interviews were invited to participate in the first survey. During the first survey, we summarized the qualitative descriptive findings from the interviews, then presented the draft consent form text for explaining each category. We informed stakeholders that our goal was to include language in the advance consent form that would be easy for patients to understand and help patients make an informed decision about participating in a noninferiority trial that uses an early enrollment strategy. We also told stakeholders that we wanted to provide concise explanations. Stakeholders reviewed the descriptive findings from the interviews and classified each statement (or group of statements) in the draft consent form text as (1) “important: remain as is,” (2) “important: but changes needed,” or (3) “unimportant: remove.” We asked stakeholders to suggest a revision if they chose response option 2 and provide a rationale if they chose option 3.

We analyzed the open-ended responses to the first survey by grouping and summarizing similar answers; we used response frequencies to assess the closed-ended responses. We used stakeholder feedback from the first survey to modify the draft consent text, and we prepared the second survey to obtain stakeholder feedback on the modified text. Only stakeholders who participated in the first survey were invited to participate in the second survey. During the second survey, stakeholders reviewed the descriptive findings from the first survey and provided feedback on the revised consent form text. They classified each statement in the draft text (or group of statements) as (1) “important: should remain” or (2) “unimportant: remove.” Stakeholders were asked to give a rationale for the removal of sentences; they could also provide overall comments for each statement or group of statements. We then reviewed the responses, using the same methods described previously, and finalized the consent text.

The Duke University Health System Institutional Review Board determined that this study meets the criteria for a declaration of exemption from further institutional review board review as described in 45 CFR 46.101(b), 45 CFR 46.102 (f), or 45 CFR 46.102 (d), and satisfies the Privacy Rule as described in 45 CFR 164.512(i).

## Results

We interviewed 52 stakeholders from June to August 2016, including 18 patients at risk of HABP/VABP and 12 caregivers of patients at risk for HABP/VABP. More than half of the patients had a bachelor’s degree or higher (10 of 18 [56%]), and all caregivers had at least a high school education. Stakeholders also included 7 investigators who conduct research on pneumonia, 5 study coordinators of pneumonia or intensive care unit–based clinical research, and 10 IRB representatives (Table 2; eTable in the Supplement).

**Table 2.** Characteristics of Patient and Caregiver Stakeholders

Characteristic	Stakeholder group, No. (%)	
	Patients (n = 18)	Caregivers (n = 12)
Age group, y		
25-44	5 (27.8)	2 (16.7)
45-64	5 (27.8)	9 (75.0)
≥65	8 (44.4)	1 (8.3)
Gender^a^		
Male	4 (22.2)	4 (33.3)
Female	14 (77.8)	8 (66.7)
Race		
Black or African American	0	2 (16.7)
White	18 (100)	10 (83.3)
Non-Hispanic/non-Latino ethnicity	18 (100)	12 (100)
Education		
Some high school (grades 9-12)	1 (5.6)	0
High school diploma or equivalent	2 (11.1)	2 (16.7)
Some college credit	3 (16.7)	3 (25.0)
Associate’s degree	2 (11.1)	3 (25.0)
Bachelor’s degree	3 (16.7)	0
Master’s degree	7 (38.9)	3 (25.0)
Doctorate or professional degree	0	1 (8.3)
Health status^b^		
Been in the ICU or had overnight hospital stay and previous HABP/VABP diagnosis or at-risk condition	17 (94.4)	12 (100)
Has chronic lung disease	16 (88.9)	6 (50.0)

Abbreviations: ICU, intensive care unit, HABP/VABP, hospital-acquired and/or ventilator-associated bacterial pneumonia.

^a^ Stakeholders were asked to report their gender identity.

^b^ Patients responded with reference to their own health status; caregivers responded with reference to the patient’s health status.

The first online survey was conducted in April and May 2017, with a response rate of 79% (n = 41), including 14 patients, 10 caregivers, 5 investigators, 4 study coordinators, and 8 IRB representatives. The second online survey was conducted from September to October 2017, with a response rate of 98% (n = 40), including 14 patients, 9 caregivers, 5 investigators, 4 study coordinators, and 8 IRB representatives.

### Three Essential Concepts

We identified 3 concepts that stakeholders stated were essential to describe in an advance consent form: (1) reassurances about patient health and treatment, (2) reasons for enrolling early, and (3) information about study antibiotics. We placed stakeholders’ suggestions about how to explain these concepts into 3 further categories for inclusion in an advance consent form: (1) reassurances on patient health and treatment, including information about study antibiotics, (2) a rationale for advance consent and early enrollment, and (3) an explanation of noninferiority. We included an explanation of noninferiority as the third category because of the importance the study team placed on explaining the concept of noninferiority to potential participants. Stakeholders’ narratives supporting these categories, including their recommendations for explaining them, are described in the next 3 sections and summarized in Table 3.

**Table 3.** Stakeholder-Suggested Essential Concepts to Explain in the Advance Consent Form, by Consent Categories

Consent category	Essential concept to explain
Reassurances on patient health and treatment	Reassurances about patient health Patient’s health is top priority Patients will be closely monitored Reassurances about treatment Patient will get treatment regardless of study participation Study antibiotics are commonly used for treating pneumonia^a^ Patients will not be given the study antibiotic if they do not develop pneumonia Treatment can be changed if not working Treatment will not be compromised due to research participation
Rationale for advance consent and early enrollment	Patients are at high risk of pneumonia Being approached for study participation does not mean patient will get pneumonia Being at high risk does not mean patient will get pneumonia Reasons patient is at high risk of pneumonia Patient currently doesn’t have pneumonia Advantages of the early enrollment strategy Making an informed decision when patient is not very ill with pneumonia Making a plan and being proactive Starting study drug earlier than with traditional consent
Noninferiority	Differences between the 2 study drugs How the study drugs will be compared during analyses

^a^ In the final consent text, this concept was described in the noninferiority category for ease of explanation.

#### Reassurances on Patient Health and Treatment

Several stakeholders said the differences between clinical care and research should be explained. Stakeholders emphasized that patients should be told their health is the top priority and their care will not be compromised if they choose to participate in the trial. Several also emphasized the importance of explaining to patients that, should they choose to take a study antibiotic, study staff will closely monitor their health to determine how well the study antibiotic is working and evaluate when they would be switched to an alternative, effective treatment for pneumonia, if the study antibiotic did not appear to be working (concept 1, Table 4).

**Table 4.** Excerpts From Stakeholders’ Narratives

Concept	Narrative excerpt
**1. Reassurances on patient health and treatment**	
Staff will closely monitor patient’s health	“Is there a point in the research…where the study drug seems to be not working? Where the physician would say, ‘Okay, we need to try a different approach?’… If they see that nothing is changing, is there a point in time when they can make a different call? For a different antibiotic or a change in the treatment plan?…Who’s going to have my back?” — Female patient, 40s
**2. Rationale for advance consent and early enrollment**	
Patients do not have pneumonia but could get it	“Clearly explain the whole process, explaining that [the consent form] isn’t saying that the patient has pneumonia or will get pneumonia. It’s just that if they do, you don’t want to unduly alarm them that thinking ‘Oh my gosh. I’m going to get pneumonia.’” —IRB chair “[Tell potential participants that] You’re at risk. You don’t have the condition yet. We don’t know if you’re going to get the condition, but you definitely have the factors that make you at high risk to get this condition. That’s why we’re approaching you.” —Investigator
Factors that place patients at risk	“I think it is important to explain what makes them at high risk, rather than just telling them that they’re at high risk.” —Study coordinator
Proactive approach	“If those decisions are made ahead of time…patients can probably make better decisions than when something very acute is happening.” —Investigator “[The consent form should say] if pneumonia were to happen, you might get pretty sick and not feel very good. You might not be in a place where you’re able to make this kind of decision… So we’re trying to be proactive and asking people, if this were to happen in the future, would you be willing to do it?” —Female patient, 60s “[The study drug] would be administered right away. There would be no waiting. There would be no, ‘We have to find somebody to sign the form.’” —Female caregiver, 60s
**3. Explanation of noninferiority**	
Limited understanding	“I don’t think most of the patients will understand the complexity of a noninferiority trial… There’s too much information. It just gets very complex for them to understand.” —IRB chair “In all fairness to the patient, a lot of doctors don’t understand what a noninferiority trial is… You’re trying to explain a very complicated concept, and whether or not, and how you would explain, would vary greatly between the patients.” —Investigator
How to explain	“I’m not sure it’s necessary to share the details of ‘we’re really trying to see if one is worse than the other’… We use the drugs in practice right now, and we think they’re essentially noninferior. We think they’re pretty similar.” —Investigator “[The consent form can state:] We want to run the clinical trial, because we want to find out the difference between A and B…is it better, are the side effects less in A than they are in B? Do they have different ones that A that B doesn’t have, that would make it easier?” —Female patient, 50s

Abbreviation: IRB, institutional review board.

Many stakeholders suggested patients should be informed that the study antibiotics are routinely used for treating pneumonia, though one is not FDA-approved for pneumonia. Several also suggested the advance consent form should state that if patients develop pneumonia, they will receive treatment regardless of whether they join the trial, and the treatment could be one of the study antibiotics or another antibiotic (that is already used as standard of care), and patients will not be given a study drug if they are not diagnosed with pneumonia.

#### Rationale for Advance Consent and Early Enrollment

Almost all stakeholders emphasized that patients should be informed that they were approached about trial participation because they are at high risk for pneumonia. Numerous stakeholders said that informing patients that they may not develop pneumonia, even though they were identified as being at risk and were invited to participate in an early enrollment clinical trial on pneumonia, was an essential concept to include. They also focused their narratives on describing that the consent form should emphasize that patients do not currently have pneumonia but that they could get it and describe the specific factors that place patients at risk for pneumonia.

Several stakeholders believed patients and their caregivers should be informed that early enrollment allows them to “make a plan” and “be proactive” in their research decision making, and provides an opportunity to make a decision about research participation before becoming sick. Some stakeholders said early enrollment allows patients to start study treatment earlier in the context of research than traditional consent because all decisions about research participation are made previously by the patient (concept 2, Table 4).

#### Explanation of Noninferiority

Most IRB representatives, investigators, and study coordinators acknowledged the difficulty of explaining noninferiority trials, and some described noninferiority trials incorrectly themselves. These stakeholders expressed concern about whether patients and caregivers would understand the concept of noninferiority. For this reason, several said the advance consent form should keep it simple in explaining the concept of noninferiority. Focus can be placed on describing the differences between the 2 study antibiotics and explaining noninferiority broadly when describing how the study antibiotics will be compared. They cautioned against including detailed design explanations, and some advised against using the word “worse” because that word could cause unwarranted concern about efficacy and the purpose of the trial.

Patients and caregivers said the explanation of how the study antibiotics are compared should focus on describing potential differences in efficacy, using words such as “about the same” or “better,” and the potential for the comparison to reveal important differences in adverse effects (concept 3, Table 4).

Several narratives demonstrated the struggle that some investigators, study coordinators, and IRB representatives had in finding an accurate description of a noninferiority hypothesis while balancing being true to the noninferiority hypothesis, avoiding unnecessary concern among potential study participants who believe they will receive an inferior drug, and acknowledging that the study antibiotics are currently used to treat pneumonia. For some, and because of misunderstanding by others, this struggle led to alternative suggestions to explain how the study drugs will be compared, such as “better,” “equivalent or similar,” “work equally well,” or “are similar within a certain range.”

### First Online Survey

eAppendix 2 in the Supplement includes frequency data from the first online survey. Most stakeholders rated almost all statements (n = 39) as “important, remain as is” or “important, but changes needed.” For statements rated as “important, but changes needed,” stakeholders suggested alternative wording for some statements and sought clarity for other statements. Some stakeholders suggested that certain sentences be removed from the consent text. For example, 12 stakeholders suggested removing the following statement about noninferiority trials: “Before this trial started, researchers determined how much less well the new drug can work compared to the existing drug and still be acceptable” Some stakeholders commented that information about noninferiority will be confusing, could scare participants, and is too detailed for a consent form. As a result of all stakeholders’ suggestions, we modified many of the sentences and removed some sentences. eAppendix 3 in the Supplement shows the revised text.

### Second Online Survey

eAppendix 4 in the Supplement includes frequency data from the second online survey. All statements (n = 38) in the revised consent text reached at least 80% consensus for “important, should remain;” 84% (n = 32) of statements reached 90% agreement. Stakeholders recommended minor edits and made a few suggestions to remove sentences, primarily to avoid repetition. Statements describing noninferiority continued to be more problematic than other categories: the percentage of statements that reached at least 90% consensus for “important, should remain” was 91% for rationale for advance consent and early enrollment, 100% for reassurances, and 74% for noninferiority. eAppendix 3 in the Supplement shows the revised final consent text.

## Discussion

The previous CTTI study reported that an early enrollment strategy using advance consent is an acceptable approach to patients and other key stakeholders in clinical research.^9^ However, the approach places novel demands on the informed consent process, particularly for a noninferiority HABP/VABP clinical trial evaluating 2 commonly prescribed antibiotics. To address this challenge, we interviewed 52 key stakeholders to identify information they would include in consent forms given this context and how to describe this information. As may be expected, stakeholders focused on providing information about the study antibiotics and the rationale for early enrollment.

Stakeholders’ comments about reassuring patients that their clinical care takes precedence over research, while an overall research ethics principle, highlights the importance of explaining in ways meaningful to potential participants how decisions about patient care are made when research is embedded in standard care settings. Consent information must also emphasize that immediate treatment will be provided regardless of research participation, so that patients will not believe that participating in research is advantageous over standard care. While such reassurances are not unique to early enrollment, they are especially important when discussing a potential serious complication such as HABP/VABP. Thus, it is imperative to reassure patients that their care will not be compromised for the purposes of facilitating research. Such information is typically not described in detail in consent forms using suggestions from key stakeholders.

Patients who are approached about trial participation before they develop the condition of interest, in this case HABP/VABP, are expected to be at higher risk for the condition, because early enrollment works best when it focuses on patients most likely to become eligible. However, informing patients about their increased risk poses a dilemma. Patients may appreciate learning their risk and the steps being taken to reduce it, but they may also experience greater anxiety from learning about a complication they had not previously considered. For this reason, researchers using advance consent, particularly for serious conditions, may wish to identify mechanisms to help patients cope with any anxiety they experience after learning of their increased risk.

A 2017 study reported that most clinical trials of infectious disease therapeutics are noninferiority trials,^8^ which allow for some loss of efficacy in the new intervention compared to older, effective standards of care in trade-off for another benefit, such as decreased adverse effects or cost.^13^ While we refer to studies that contain noninferiority hypotheses as “noninferiority trials,” the inherent trade-off means that trials with noninferiority hypotheses should also have superiority hypotheses associated with proposed non-efficacy benefits, such as decreases in adverse effects. Hypothesized superiority of non-efficacy benefits is necessary to justify the choice of the noninferiority hypothesis and to explain the proposed trade-off between potential lesser efficacy and non-efficacy benefits. In our example, we posed that meropenem might have fewer adverse events than imipenem in trade-off for a potential loss of effect.

Researchers have described the need for different informed consent language for noninferiority trials.^14^ Yet, multiple interpretations of the basic principles of noninferiority remain,^15^ as suggested in our results. While there were concerns by stakeholders that patients might not understand the noninferiority concept, some investigators, study coordinators, and IRB members also had their own misconceptions. Others have documented a lack of varied understandings of noninferiority trials, including the trade-offs.^16^ We also found that whether and how to explain a noninferiority trial in consent text eludes easy consensus. Ultimately, this confusion and concern might reflect that the noninferiority design is not an intuitive statistical or clinical concept, whereas superiority designs are likely more readily understandable or more familiar. Nevertheless, inscrutability cannot be the rationale for not explaining important trial design aspects to potential participants, as explaining the study purpose is required by ethics guidelines (45 CFR 46.116).^12^ Consent text should emphasize the potential trade-offs between antibiotic efficacy and other benefits, acknowledging that a trial result of lesser efficacy may be considered a successful outcome. Others are engaging patients in the selection of noninferiority margins and the inherent trade-offs in these trials.^17^

### Strengths and Limitations

The strengths of our study include input from diverse stakeholders and a use of an iterative process for soliciting and incorporating their feedback. Other researchers have reported the need for patient input in designing clinical trials.^17^ Although the proposed consent language was developed for a HABP/VABP trial, the language can be adapted for other noninferiority trials and trials using an early enrollment strategy.

Our study also has limitations. First, we used purposive sampling to select patients similar to those enrolled in HABP/VABP trials, and their opinions may not be the same as other patient populations, particularly given that many of these stakeholders had high levels of education. Second, we did not evaluate the final proposed consent language after the second online survey. Further research can assess the utility of this language with a larger number of stakeholders, including how best to modify the language for non-US populations. We do not intend, however, for the proposed language to be used verbatim in all situations. Rather, we hope the language will inform researchers’ decisions about the kinds of information to include in consent documents and provide sample text that is acceptable to many stakeholders. Third, we were guided by stakeholders’ statements but made necessarily subjective judgments about what to include and how to phrase the statements. Fourth, we explained early enrollment and noninferiority (for some stakeholders) at the beginning of the qualitative interview, which may have informed stakeholders’ suggestions for topics to include in the consent text. Fifth, consent text for other noninferiority trials will change based on the hypothesized non-efficacy benefits proposed with the new intervention.

Adding language to already lengthy consent forms may be undesirable. However, every sentence in the final consent text was rated as “important, should remain” by most stakeholders. We envision that this sample language will replace some current sections of consent forms while providing greater clarity on study purpose and potential harms and benefits for potential study participants, which is a goal of informed consent.

## Conclusions

We used data-driven input from key stakeholders to generate proposed consent language for noninferiority trials using an early enrollment strategy. The proposed consent language, in combination with a strategy for enrolling patients at highest risk for pneumonia, may help potential participants make informed decisions about their involvement in the clinical research and improve enrollment rates that are necessary to evaluate new treatments and improve patient care.

## Notes

Supplement. **eAppendix 1.** Information Provided to Stakeholders at the Beginning of the Qualitative Interview to Provide Context for the Noninferiority Pilot Clinical Trial Using an Early Enrollment Approach **eTable.** Characteristics of Investigator, Coordinator, and IRB Representative Stakeholders **eReference.** **eAppendix 2.** Responses to Online Survey 1 **eAppendix 3.** Advance Informed Consent Form Text **eAppendix 4.** Responses to Online Survey 2
