# Supplementary material for: Consensus on Language for Advance Informed Consent in Health Care–Associated Pneumonia Clinical Trials Using a Delphi Process
Source: JAMA Netw Open. 2020 May 22;3(5):e205435. doi: 10.1001/jamanetworkopen.2020.5435 (PMC7244987; doi:10.1001/jamanetworkopen.2020.5435)
Supplement: Supplement. — eAppendix 1. Information Provided to Stakeholders at the Beginning of the Qualitative Interview to Provide Context for the Noninferiority Pilot Clinical Trial Using an Early Enrollment Approach eTable. Characteristics of Investigator, Coordinator, and IRB Representative Stakeholders eReference. eAppendix 2. Responses to Online Survey 1 eAppendix 3. Advance Informed Consent Form Text eAppendix 4. Responses to Online Survey 2 [file jamanetwopen-3-e205435-s001.pdf]

Supplementary Online Content

Corneli A, Calvert SB, Powers JH III, et al. Consensus on language for advance informed consent in health care–associated pneumonia clinical trials using a Delphi process. *JAMA Netw Open*. 2020;3(5):e205435. doi:10.1001/jamanetworkopen.2020.5435

eAppendix 1. Information Provided to Stakeholders at the Beginning of the Qualitative Interview to Provide Context for the Noninferiority Pilot Clinical Trial Using an Early Enrollment Approach
eTable. Characteristics of Investigator, Coordinator, and IRB Representative Stakeholders
eReference
eAppendix 2. Responses to Online Survey 1
eAppendix 3. Advance Informed Consent Form Text
eAppendix 4. Responses to Online Survey 2

This supplementary material has been provided by the authors to give readers additional information about their work.

© 2020 Corneli A et al. *JAMA Network Open.*

eAppendix 1: Information provided to stakeholders at the beginning of the qualitative interview to provide context for the noninferiority pilot clinical trial using an early enrollment approach.

Patients and caregivers viewed pictorial cue cards during the interview that matched the verbal explanation of the study described below.

1. Information provided to patients and caregivers

I now want to tell you more about the early enrollment study. This will take a bit of time as I want to make sure I explain everything clearly to you. I also want to make sure that you understand this information, as some of it is tricky to explain. So please feel free to interrupt me at any time if I say something that is not clear.

Today we will talk about clinical trials to find new antibiotics for treating people with pneumonia. Pneumonia is a type of lung infection. We will focus on pneumonias that people can get when they are in the hospital or on a machine called a ventilator, which helps people to breathe.

Patients can agree to take part in clinical trials in two ways. One way is where a patient personally agrees to take part in the trial, after learning all about the trial. The other way is for the patient's legally authorized representative to agree for the patient to take part in the trial. A legally authorized representative is someone who can legally speak for the patient. This is done when patients are unable to provide their own consent.

It is hard to find patients who are able to provide their own informed consent to take part in clinical trials on new antibiotics for pneumonia. Often these patients feel very sick and may not be able to give their consent. When patients cannot give their consent on their own, it is often hard to find the patients' legally authorized representative so that person can provide consent for the patient, if they have a legally authorized representative.

For these reasons, researchers at CTTI are trying a new approach to increase the number of patients who can enroll in clinical trials on pneumonia. They want to find out if enrolling patients *early* into a clinical trial can lead to more people taking part in the trial. Enrolling patients early means that patients who are at high risk for developing pneumonia while in the hospital would join the trial *before* they develop pneumonia. By saying a patient is at "high risk," it means that they are in a situation that makes them more likely than others to develop pneumonia, although they may not develop pneumonia. This can happen when a patient is in the hospital for longer than 48 hours or if they are on a machine to help them breathe.

Researchers at CTTI plan to study this new early enrollment approach to find out if it allows more patients to choose to enroll in clinical trials on pneumonia. When patients join that study, they agree to two parts. First, they agree that study staff can access their hospital records during their stay in the ICU. This is so they can see if the patient develops pneumonia. Second, if patients do develop pneumonia, they agree to receive one of two antibiotics as part of the trial. What's new

© 2020 Corneli A et al. *JAMA Network Open.*

about this approach is that patients consent for these two parts *before* they develop pneumonia. What usually happens is that patients are asked to join a clinical trial only after they have already developed pneumonia.

So during the interview today, I want to hear what you think of this early enrollment approach. But before we continue, do you have any questions about this early enrollment approach?

Based on the information I provided you, what is your understanding of this approach? [Interviewer: repeat information as necessary.]

Ok, let's continue. Now I will tell you more about the second part of the future study. If a patient enrolls in the study and develops pneumonia, he or she would be placed into one of two groups by chance. Patients in one group will get Antibiotic A. Patients in the other group will get Antibiotic B. Both antibiotics were approved many years ago by the U.S. Food and Drug Administration to treat infections. I'll refer to the U.S. Food and Drug Administration as FDA from now on.

It's important to know that Antibiotic A has been approved by the FDA to treat pneumonia but Antibiotic B has not. Antibiotic B has only been approved by the FDA to treat other serious infections. What this means is that Antibiotic A was tested in a clinical trial and shown to work in treating pneumonia. It was then approved by the FDA for the treatment of pneumonia. Antibiotic B was tested in clinical trials on other serious infections and shown to work for those infections. It was then approved by the FDA for the treatment of those infections. While Antibiotic B has not been approved by the FDA for the treatment of pneumonia, it is commonly used in hospitals throughout the United States as a treatment for pneumonia. This is called "off-label use" and it's commonly done with many drugs. Researchers now want to test the antibiotics that are used in that way so they know for sure how well they work in treating these other infections. This is because antibiotics act in different ways with different patients and different infections.

[Interviewer: Before continuing on, ask the participant if she/he has any questions and to restate her/his understanding of the two study drugs; provide additional information as needed.]

Ok, let's continue. Even though Antibiotic A and Antibiotic B have been commonly used by physicians to treat pneumonia for years, CTTI researchers want to find out more about Antibiotic B. A clinical trial is the best way to learn about Antibiotic B. This is part 2 of the future study. Patients who enroll in this future study should know that Antibiotic B might not work as well as Antibiotic A in treating pneumonia. However, it might have fewer side effects. The clinical trial will find this out.

The last point I want to make is that if patients choose not to join this trial, they would get the antibiotics the hospital typically uses to treat pneumonia, if they did develop pneumonia. Because Antibiotic A and Antibiotic B are commonly used in hospitals to treat pneumonia, patients who don't join the trial may still get either Antibiotic A or Antibiotic B. But, they may also get another drug which is not Antibiotic A or Antibiotic B.

© 2020 Corneli A et al. *JAMA Network Open.*

For patients who join this study but don't develop pneumonia, their part in this study will end when they leave the hospital.

[Interviewer: Before continuing on, ask the participant if she/he has any questions and to restate her/his understanding of the reason a clinical trial is needed in this situation and standard-of-care; provide additional information as needed.]

We are not asking you to join that study today. The point of the interview today is to hear what you think is acceptable and not acceptable about the new early enrollment approach the CTTI researchers want to try. We also want to find out how we can best explain this study to patients.

2. Information provided to investigators and study coordinators

Investigators are interested in assessing whether an early enrollment strategy can improve enrollment rates for clinical trials on hospital-acquired bacterial pneumonia and ventilator-associated bacterial pneumonia, referred to as HABP/VABP. Using this strategy, patients at high risk for developing HABP or VABP, or their legally authorized representative, are asked to give their informed consent to enroll in a clinical trial in advance of developing HABP/VABP. Patients agree to study staff monitoring their condition, and if pneumonia develops, to being randomized to receive one of the study drugs.

Planning has begun on a pilot study to evaluate the effectiveness of this early enrollment strategy. For the findings to be applicable to future investigational clinical trials, the early enrollment study must be embedded within a treatment trial. Patients must not only consider whether they are willing to enroll in a trial before they develop pneumonia, but also whether they are willing be randomized to one of the study drugs for the purpose of answering a treatment question, if they do develop pneumonia. Therefore, in the early enrollment study, patients will be asked to consider enrolling early in a trial where they will be randomized to receive either imipenem or meropenem, if they develop pneumonia. A secondary objective of the study is to identify whether meropenem is not worse in treating HABP/VABP than imipenem by more than a pre-specified amount and to identify any differences in adverse events between the two drugs. This is referred to as a noninferiority trial.

Both drugs were approved many years ago by the FDA to treat infections. Imipenem has been approved by the FDA to treat lower respiratory tract infection, which is pneumonia, but meropenem has not. Meropenem has only been approved by the FDA to treat other serious infections. However, meropenem is commonly used off-label in hospitals throughout the United States as a treatment for pneumonia.

3. Information provided to IRB representatives

Investigators are interested in assessing whether an early enrollment strategy can improve enrollment rates for clinical trials on hospital-acquired bacterial pneumonia and ventilator-

associated bacterial pneumonia, referred to as HABP/VABP. Using this strategy, patients at high risk for developing HABP or VABP while in the hospital, or their legally authorized representatives, are asked to give their informed consent to enroll in a clinical trial in advance of developing an infection. By providing their consent to join the study, they agree to two activities. First, patients agree that study staff can access their hospital records while they are in the hospital to see if they develop pneumonia. Second, if pneumonia develops, they agree to being randomized to receive one of the study drugs. At the time of infection, the patient can opt out of being randomized.

Researchers at CTTI have submitted a protocol for a pilot study to evaluate the effectiveness of this early enrollment strategy. This will be a two-arm study, with initial randomization at the site level. The control arm is standard enrollment at the time of infection and the intervention arm is early enrollment prior to infection. For the findings to be applicable to future investigational clinical trials, the evaluation must be embedded within a treatment trial. For this trial, patient will be randomized to receive one of two drugs. Therefore, patients must not only consider whether they are willing to enroll in a trial before they develop pneumonia, but also whether they are willing be randomized to one of the study drugs for the purpose of answering a treatment question, if they do develop pneumonia. Therefore, in the early enrollment study, patients will be asked to consider enrolling early in a trial where they will be randomized to receive either imipenem or meropenem, if they develop pneumonia. A secondary objective of the study is to identify whether meropenem is not worse in treating HABP/VABP than imipenem by more than a pre-specified amount and to identify any differences in adverse events between the two antibiotics.

Both antibiotics were approved many years ago by the FDA to treat infections. Imipenem has been approved by the FDA to treat lower respiratory tract infection, which is pneumonia, but meropenem has not. Meropenem has only been approved by the FDA to treat other serious infections. However, meropenem is commonly used off-label in hospitals throughout the United States as a treatment for pneumonia.

© 2020 Corneli A et al. *JAMA Network Open.*

eTable. Characteristics of Investigator, Study Coordinator, and IRB Representative Stakeholders

Characteristic	No. (%)
Investigators (n = 7)	
Investigator in the PROPHETIC study[a]	7 (100.0)
Study coordinators (n = 5)	
Years of experience	
1-3	2 (40.0)
4-5	2 (40.0)
More than 5	1 (20.0)
IRB representatives (n = 10)	
Role	
Chairperson	6 (60.0)
Scientific member	2 (20.0)
Community member	2 (20.0)
Years of experience	
1-5	2 (20.0)
6-10	3 (30.0)
11-15	2 (20.0)
16-20	3 (30.0)

Abbreviation: IRB, institutional review board.
[a]The PROPHETIC study is a prospective observational cohort study conducted at 28 intensive care units (ICUs) in the United States to determine the proportion of at-risk patients who develop pneumonia.[1]

© 2020 Corneli A et al. *JAMA Network Open.*

eReference

1. Bergin S, Coles A, Farley J, et al. Predicting pneumonia: a prospective observational study of the risk factors for hospital-acquired and ventilator-associated bacterial pneumonia. Am J Respir Crit Care Med. 2017;195:A2642.

eAppendix 2: Responses to Online Survey #1

This eAppendix provides the frequency data for each statement or group of statements assessed by stakeholders during the first online survey.

Stakeholders could choose one of the following three responses for each statement or group of statements: (1) “Important: Remain as is”, (2) “Important, but changes needed”, and 3) “Unimportant: Remove”. Stakeholders were asked to provide their rationale in an open-ended field if they selected “Important, but changes needed” or “Unimportant: Remove”

For the response, “Important, but changes needed”:

1. We re-coded stakeholder responses and placed them in their own category when, in the open-ended field, stakeholders:

    a. Provided comments that were unrelated to the statement or group of statements (such as describing their own experiences or suggesting a topic addressed elsewhere)
    b. Suggested no re-wording of the statement or group of statements
    c. Asked questions or provided comments about the study that were unrelated any perceived ambiguities within the statement or group of statements

2. We kept responses as reported (i.e., did not re-code) in situations when only one word change was suggested (for example, one stakeholder suggested that “patients” should be changed to “subjects”; another stakeholder suggested that “the clinical trial” be changed to “this clinical trial”).

For the response, “Unimportant: Remove”:

1. We recoded stakeholder responses and moved them to “Important, but changes needed” when the stakeholder suggested text changes in the open-ended field.

2. We kept the response as reported (i.e., did not re-code) in situations when the stakeholder suggested to remove only a portion of a statement.

All re-coding was verified by two analysts.

© 2020 Corneli A et al. *JAMA Network Open.*

Category #1: Rationale for advance consent and early enrollment

A. **Number of stakeholder respondents and response rate**

Stakeholder Group	n (%)
Patients	14 (78)
LARs	10 (83)
Coordinators	4 (80)
Investigators	5 (71)
IRB	8 (80)
Total completion	41 (79)

B. **Statements or groups of statements**

1. **Statement: We are trying a new way of enrolling patients into clinical trials for treating pneumonia. This new way is to enroll patients into the trial before they get pneumonia.**

Response Option	Stakeholder Group (n)					Total
	Patients	LARs	Coordinators	Investigators	IRB	
Important: Remain as is	12	9	3	5	6	35
Important, but changes needed	0	1	1	0	2	4
Recoded from "Important, but changes needed"	2	0	0	0	0	2
Unimportant: Remove	0	0	0	0	0	0

2. **Statement: Patients who will be enrolled are in the intensive care unit and are at high risk for getting pneumonia while in the hospital.**

Response Option	Stakeholder Group (n)					Total
	Patients	LARs	Coordinators	Investigators	IRB	
Important: Remain as is	10	9	4	5	7	35
Important, but changes needed	2	1	0	0	1	4
Recoded from "Important, but changes needed"	1	0	0	0	0	1
Unimportant: Remove	1	0	0	0	0	1

3. **Statement: We want to find out if enrolling patients early like this can lead to more patients taking part in clinical trials and can speed up the process of developing new medicines for patients in the future.**

Response Option	Stakeholder Group (n)					Total
	Patients	LARs	Coordinators	Investigators	IRB	
Important: Remain as is	9	7	3	2	4	25
Important, but changes needed	4	1	1	2	4	12
Recoded from "Important, but changes needed"	0	2	0	0	0	2
Unimportant: Remove	1	0	0	1	0	2

© 2020 Corneli A et al. *JAMA Network Open.*

4. **Statement: You do not have pneumonia now. We are talking to you because you have a greater chance of getting pneumonia compared to other patients who are not in the intensive care unit. This means that there is a chance that you could get pneumonia while you are in the hospital.**

Response Option	Stakeholder Group (n)					Total
	Patients	LARs	Coordinators	Investigators	IRB	
Important: Remain as is	12	10	3	4	6	35
Important, but changes needed	1	0	1	1	1	4
Recoded from "Important, but changes needed"	0	0	0	0	1	1
Unimportant: Remove	1	0	0	0	0	1

5. **Statement: The reasons you might get pneumonia are because you may have been in the hospital for longer than 48 hours or are on a machine that helps you to breathe.**

Response Option	Stakeholder Group (n)					Total
	Patients	LARs	Coordinators	Investigators	IRB	
Important: Remain as is	11	7	4	3	6	31
Important, but changes needed	3	3	0	1	2	9
Recoded from "Important, but changes needed"	0	0	0	1	0	1
Unimportant: Remove	0	0	0	0	0	0

6. **Statement: There may also be other reasons that put you at a greater chance of getting pneumonia. Your doctor can tell you those reasons.**

Response Option	Stakeholder Group (n)					Total
	Patients	LARs	Coordinators	Investigators	IRB	
Important: Remain as is	11	3	3	3	6	26
Important, but changes needed	1	5	1	2	1	10
Recoded from "Important, but changes needed"	0	1	0	0	0	1
Unimportant: Remove	2	1	0	0	1	4

7. **Statement: If you choose to join this study, you will be agreeing to two parts. First, you agree that study staff can access your hospital records while you are in the hospital. This is so they can see if you get pneumonia during this time. Second, if you do get pneumonia, you agree to receive one of the two study antibiotics that are being tested as part of the trial.**

Response Option	Stakeholder Group (n)					Total*
	Patients	LARs	Coordinators	Investigators	IRB	
Important: Remain as is	10	9	2	4	7	32
Important, but changes needed	2	0	2	1	0	5
Recoded from "Important, but changes needed"	2	1	0	0	0	3
Unimportant: Remove	0	0	0	0	0	0

*A response from an IRB representative is missing.

© 2020 Corneli A et al. *JAMA Network Open.*

8. **Statement: What is new about this approach is that you are being asked to consent for these two parts while you are only at risk for getting pneumonia, meaning there is a chance you could get pneumonia. In most trials, you would be asked to join the study only after you had already gotten pneumonia.**

Response Option	Stakeholder Group (n)					Total
	Patients	LARs	Coordinators	Investigators	IRB	
Important: Remain as is	8	10	3	3	6	30
Important, but changes needed	4	0	1	2	2	9
Recoded from "Important, but changes needed"	1	0	0	0	0	1
Unimportant: Remove	1	0	0	0	0	1

9. **Statement: We want to make sure that you understand the clinical trial and make your own decision about taking part.**

Response Option	Stakeholder Group (n)					Total
	Patients	LARs	Coordinators	Investigators	IRB	
Important: Remain as is	13	10	4	5	6	38
Important, but changes needed	1	0	0	0	2	3
Unimportant: Remove	0	0	0	0	0	0

10. **Statement: If you are unable to make a decision on your own at this time, we want to make sure your legally authorized representative (LAR), if you have one, understands the clinical trial so she/he can make a decision on your behalf.**

Response Option	Stakeholder Group (n)					Total
	Patients	LARs	Coordinators	Investigators	IRB	
Important: Remain as is	13	7	2	3	3	28
Important, but changes needed	0	2	2	0	3	7
Recoded from "Important, but changes needed"	1	0	0	1	0	2
Unimportant: Remove	0	1	0	1	2	4

11. **Statement: By telling you (and/or your LAR) about the clinical trial now, you have more time to decide if you want to take part.**

Response Option	Stakeholder Group (n)					Total
	Patients	LARs	Coordinators	Investigators	IRB	
Important: Remain as is	11	8	4	5	6	34
Important, but changes needed	1	2	0	0	2	5
Recoded from "Important, but changes needed"	2	0	0	0	0	2
Unimportant: Remove	0	0	0	0	0	0

© 2020 Corneli A et al. *JAMA Network Open.*

12. **Statement: Also, if you get pneumonia later and are asked to think about taking part in the trial at that time, it may be harder to make a decision because you may feel very sick.**

Response Option	Stakeholder Group (n)s					Total
	Patients	LARs	Coordinators	Investigators	IRB	
Important: Remain as is	10	9	4	4	7	34
Important, but changes needed	4	1	0	0	1	6
Recoded from "Important, but changes needed"	0	0	0	1	0	1
Unimportant: Remove	0	0	0	0	0	0

13. **Statement: If you decide to not join the trial and you get pneumonia, you will be given the standard antibiotic that the hospital uses to treat pneumonia.**

Response Option	Stakeholder Group (n)					Total
	Patients	LARs	Coordinators	Investigators	IRB	
Important: Remain as is	13	8	3	4	8	36
Important, but changes needed	0	2	1	1	0	4
Unimportant: Remove	1	0	0	0	0	1

14. **Statement: This might make you unable to take part in the clinical trial because the Food and Drug Administration (FDA) has a rule that patients cannot join pneumonia studies if they have been treated with an antibiotic for more than 24 hours.**

Response Option	Stakeholder Group (n)					Total
	Patients	LARs	Coordinators	Investigators	IRB	
Important: Remain as is	9	9	4	4	5	31
Important, but changes needed	1	0	0	0	1	2
Recoded from "Important, but changes needed"	3	0	0	0	0	3
Unimportant: Remove	1	1	0	1	2	5

15. **Statement: If you decide now that you do want to take part in the clinical trial, then you will be given one of the two study antibiotics if you do get pneumonia.**

Response Option	Stakeholder Group (n)					Total
	Patients	LARs	Coordinators	Investigators	IRB	
Important: Remain as is	12	10	2	4	7	35
Important, but changes needed	1	0	2	1	1	5
Unimportant: Remove	1	0	0	0	0	1

© 2020 Corneli A et al. *JAMA Network Open.*

16. **Statement: It is also possible that you will NOT get pneumonia. If you agree to take part in this clinical trial and do not get pneumonia, you will not need treatment for pneumonia. You will not get either of the study antibiotics.**

Response Option	Stakeholder Group (n)					Total
	Patients	LARs	Coordinators	Investigators	IRB	
Important: Remain as is	10	10	3	5	8	36
Important, but changes needed	2	0	1	0	0	3
Recoded from “Important, but changes needed	1	0	0	0	0	1
Unimportant: Remove	1	0	0	0	0	1

© 2020 Corneli A et al. *JAMA Network Open.*

Category #2: Reassurances

A. **Number of stakeholder respondents and response rate**

Stakeholder Group	n (%)
Patients	12 (67)
LARs	10 (83)
Coordinators	4 (80)
Investigators	5 (71)
IRB	8 (80)
Total completion	39 (75)

B. **Statements or groups of statements**

1. **Statement: People who get pneumonia when in the hospital are very sick. Some of these patients do not get better and may die, despite taking medicines.**

Response Option	Stakeholder Group (n)					Total
	Patients	LARs	Coordinators	Investigators	IRB	
Important: Remain as is	9	9	2	5	8	33
Important, but changes needed	2	1	2	0	0	5
Recoded from “Important, but changes needed	1	0	0	0	0	1
Unimportant: Remove	0	0	0	0	0	0

2. **Statement: If you decide to join this clinical trial, you will be very closely watched to see if you get pneumonia.**

Response Option	Stakeholder Group (n)					Total
	Patients	LARs	Coordinators	Investigators	IRB	
Important: Remain as is	10	9	4	5	8	36
Important, but changes needed	2	0	0	0	0	2
Unimportant: Remove	0	1	0	0	0	1

3. **Statement: If you do get pneumonia and take a study drug, you will continue to be very closely watched.**

Response Option	Stakeholder Group (n)					Total
	Patients	LARs	Coordinators	Investigators	IRB	
Important: Remain as is	10	10	4	5	8	37
Important, but changes needed	2	0	0	0	0	2
Unimportant: Remove	0	0	0	0	0	0

© 2020 Corneli A et al. *JAMA Network Open.*

4. Statement: Your health is the top priority of your care team and the study investigator.

Response Option	Stakeholder Group (n)					Total
	Patients	LARs	Coordinators	Investigators	IRB	
Important: Remain as is	10	10	4	5	8	37
Important, but changes needed	1	0	0	0	0	1
Unimportant: Remove	1	0	0	0	0	1

5. Statement: Study staff will pay close attention to how you are responding to the study drug.

Response Option	Stakeholder Group (n)					Total*
	Patients	LARs	Coordinators	Investigators	IRB	
Important: Remain as is	9	9	2	4	7	31
Important, but changes needed	2	0	2	1	0	5
Unimportant: Remove	0	0	0	0	1	1

*A response from patient and a LAR is missing.

6. Statement: If it appears that the study drug is not working for you, then you will be given a different drug to try to treat your pneumonia.

Response Option	Stakeholder Group (n)					Total*
	Patients	LARs	Coordinators	Investigators	IRB	
Important: Remain as is	6	7	3	2	3	21
Important, but changes needed	5	2	1	3	5	16
Unimportant: Remove	0	0	0	0	0	0

*A response from a patient and a LAR is missing.

© 2020 Corneli A et al. *JAMA Network Open.*

Category #3: Noninferiority

A. **Number of stakeholder respondents and response rate**

Stakeholder Group	n (%)
Patients	13 (72)
LARs	10 (83)
Coordinators	4 (80)
Investigators	5 (71)
IRB	8 (80)
Total completion	40 (77)

B. **Statements or groups of statements**

1. **Statement: What is the reason for this clinical trial? We want to find new drugs that treat the types of pneumonia that people get when in the hospital.**

Response Option	Stakeholder Group (n)					Total
	Patients	LARs	Coordinators	Investigators	IRB	
Important: Remain as is	12	10	3	5	8	38
Important, but changes needed	1	0	0	0	0	1
Unimportant: Remove	0	0	1	0	0	1

2. **Statement: This is because patients need more medication options. Some patients cannot take certain drugs. Also, some drugs might not work as well as other drugs but have benefits that patients want.**

Response Option	Stakeholder Group (n)					Total
	Patients	LARs	Coordinators	Investigators	IRB	
Important: Remain as is	11	9	3	4	5	32
Important, but changes needed	1	1	0	1	3	6
Recoded from “Important, but changes needed	1	0	0	0	0	1
Unimportant: Remove	0	0	1	0	0	1

3. **Statement: For example, some drugs work very well in treating pneumonia but have severe side effects. Patients who want to limit side effects may want another drug that has fewer side effects, even if it may treat pneumonia less well.**

Response Option	Stakeholder Group (n)					Total
	Patients	LARs	Coordinators	Investigators	IRB	
Important: Remain as is	8	9	3	3	5	28
Important, but changes needed	2	1	0	1	2	6
Recoded from “Important, but changes needed	2	0	0	0	0	2
Unimportant: Remove	1	0	1	1	1	4

4. **Statement: To be able to have more treatment options, clinical trials must be done to find out if new drugs can treat pneumonia and are safe.**

Response Option	Stakeholder Group (n)					Total
	Patients	LARs	Coordinators	Investigators	IRB	
Important: Remain as is	11	10	3	5	8	37
Important, but changes needed	2	0	1	0	0	3
Unimportant: Remove	0	0	0	0	0	0

5. **Statement: When more than one drug is available, patients can choose the trade-offs that work best for them.**

Response Option	Stakeholder Group (n)					Total
	Patients	LARs	Coordinators	Investigators	IRB	
Important: Remain as is	11	10	2	2	3	28
Important, but changes needed	0	0	1	3	5	9
Unimportant: Remove	2	0	1	0	0	3

6. **Statement: What is the purpose of this clinical trial? The purpose of this clinical trial is to find out if a new drug can treat the types of pneumonia that people get in the hospital.**

Response Option	Stakeholder Group (n)					Total
	Patients	LARs	Coordinators	Investigators	IRB	
Important: Remain as is	9	9	2	5	8	33
Important, but changes needed	3	0	2	0	0	5
Recoded from “Important, but changes needed	1	0	0	0	0	1
Unimportant: Remove	0	1	0	0	0	1

7. **Statement: To do this, researchers must test how well the new drug works in treating pneumonia, compared to an existing drug that has already been shown to treat this type of pneumonia.**

Response Option	Stakeholder Group (n)					Total
	Patients	LARs	Coordinators	Investigators	IRB	
Important: Remain as is	12	10	2	5	8	37
Important, but changes needed	1	0	1	0	0	2
Unimportant: Remove	0	0	1	0	0	1

8. **Statement: Researchers believe that the new drug can treat pneumonia, but it is possible that it may work less well than the existing drug.**

Response Option	Stakeholder Group (n)					Total
	Patients	LARs	Coordinators	Investigators	IRB	
Important: Remain as is	11	10	3	2	4	30
Important, but changes needed	2	0	0	2	3	7
Unimportant: Remove	0	0	1	1	1	3

© 2020 Corneli A et al. *JAMA Network Open.*

9. **Statement: Before this trial started, researchers determined how much less well the new drug can work compared to the existing drug and still be acceptable.**

Response Option	Stakeholder Group (n)					Total
	Patients	LARs	Coordinators	Investigators	IRB	
Important: Remain as is	6	6	2	2	2	18
Important, but changes needed	2	2	0	2	3	9
Recoded from “Important, but changes needed	1	0	0	0	0	1
Unimportant: Remove	4	2	2	1	3	12

10. **Statement: Researchers believe the difference between the two drugs will likely not impact patient’s overall health.**

Response Option	Stakeholder Group (n)					Total
	Patients	LARs	Coordinators	Investigators	IRB	
Important: Remain as is	12	8	2	3	5	30
Important, but changes needed	1	2	1	1	1	6
Unimportant: Remove	0	0	1	1	2	4

11. **Statement: The existing drug is called [insert name]. It has been approved by the U.S. Food and Drug Administration (FDA) for the treatment of pneumonia.**

Response Option	Stakeholder Group (n)					Total
	Patients	LARs	Coordinators	Investigators	IRB	
Important: Remain as is	13	10	4	5	8	40
Important, but changes needed	0	0	0	0	0	0
Unimportant: Remove	0	0	0	0	0	0

12. **Statement: The new drug is called [insert name]. It is considered "new"� because it has not been approved by the FDA for the treatment of pneumonia. However, it has been approved by the FDA for the treatment of [insert condition(s)]. This means that researchers know some information about how this drug affects people.**

Response Option	Stakeholder Group (n)					Total*
	Patients	LARs	Coordinators	Investigators	IRB	
Important: Remain as is	9	8	2	4	7	30
Important, but changes needed	2	1	2	1	1	7
Recoded from “Important, but changes needed	1	0	0	0	0	1
Unimportant: Remove	1	0	0	0	0	1

*A response from a LAR is missing.

© 2020 Corneli A et al. *JAMA Network Open.*

13. **Statement: For example, researchers know some of side effects to expect when this drug is used. The reason researchers feel the new drug is worth testing is because it may offer benefits to patients that the existing drug does not.**

Response Option	Stakeholder Group (n)					Total
	Patients	LARs	Coordinators	Investigators	IRB	
Important: Remain as is	11	10	3	5	7	36
Important, but changes needed	1	0	0	0	1	2
Unimportant: Remove	1	0	1	0	0	2

14. **Statement: The best way to find out if the new drug works in treating pneumonia and has any benefits compared to the existing drug is to do the clinical trial.**

Response Option	Stakeholder Group (n)					Total
	Patients	LARs	Coordinators	Investigators	IRB	
Important: Remain as is	11	9	3	5	8	36
Important, but changes needed	1	0	1	0	0	2
Unimportant: Remove	1	1	0	0	0	2

15. **Statement: For patients in this clinical trial who get pneumonia, some will get the new drug by chance and some will get the existing drug by chance.**

Response Option	Stakeholder Group (n)					Total
	Patients	LARs	Coordinators	Investigators	IRB	
Important: Remain as is	10	8	3	4	6	31
Important, but changes needed	2	1	1	1	2	7
Unimportant: Remove	1	1	0	0	0	2

16. **Statement: At the end of the trial, the results between the two groups of patients will be compared.**

Response Option	Stakeholder Group (n)					Total*
	Patients	LARs	Coordinators	Investigators	IRB	
Important: Remain as is	12	10	4	3	7	36
Important, but changes needed	1	0	0	1	0	2
Unimportant: Remove	0	0	0	1	0	1

*A response from an IRB representative is missing.

© 2020 Corneli A et al. *JAMA Network Open.*

17. **Statement: If the new drug works less well than the existing drug in treating pneumonia, by more than the amount specified before the trial, researchers will conclude that the new drug does not work in treating pneumonia.**

**Response Option**	**Stakeholder Group (n)**					**Total**
	**Patients**	**LARs**	**Coordinators**	**Investigators**	**IRB**	
Important: Remain as is	12	8	3	2	5	30
Important, but changes needed	1	1	0	0	3	5
Recoded from “Important, but changes needed	0	0	0	1	0	1
Unimportant: Remove	0	1	1	2	0	4

© 2020 Corneli A et al. *JAMA Network Open.*

eAppendix 3: Advance informed consent form text.

Of note: For the first online survey, prior to reading the draft informed consent text, stakeholders could choose 1 of 2 ways to learn the qualitative descriptive findings for each consent category—either by listening to an embedded audio-visual presentation or by reading a written version of the presentation. For the second online survey, stakeholders read the descriptive findings from the first survey before reading the revised draft informed consent text.

A. **Text on reassurances on patient health and treatment.**

1. **Original text developed using qualitative interview findings. Presented during online survey #1.**

People who get pneumonia when in the hospital are very sick. Some of these patients do not get better and may die, despite taking medicines. If you decide to join this clinical trial, you will be very closely watched to see if you get pneumonia. If you do get pneumonia and take a study drug, you will continue to be very closely watched. Your health is the top priority of your care team and the study investigator. Study staff will pay close attention to how you are responding to the study drug. If it appears that the study drug is not working for you, then you will be given a different drug to try to treat your pneumonia.

2. **Revised text based on online survey #1 findings. Presented during online survey #2.**

People who get pneumonia when in the hospital are very sick. Some of these patients do not get better and may die, despite taking medicines. This can happen to patients who join this clinical trial and to patients who do not.

If you decide to join this clinical trial, you will be very closely watched by both your healthcare team and by trial staff to see if you get pneumonia. If you do get pneumonia and take one of the antibiotics used in the trial, you will continue to be very closely watched. Your health is the top priority of your healthcare team and trial staff. They will pay close attention to how you respond to the antibiotic you received as part of the trial. If your healthcare team and trial staff feel the antibiotic is not working for you after [add period of time], you will be given a different antibiotic to try to treat your pneumonia. These will be the standard antibiotics your healthcare team normally uses at this hospital to treat pneumonia.

If you decide to NOT join this clinical trial, you will be watched closely by your healthcare team to see if you get pneumonia. If you do get pneumonia, you will be given the standard antibiotics that your healthcare team normally uses at this hospital to treat pneumonia. Your healthcare team will watch you closely and monitor how you are responding to any treatment. Your health is the top priority of your healthcare team.

© 2020 Corneli A et al. *JAMA Network Open.*

3. Final proposed text.

People who get pneumonia when in the hospital are very sick. Some of these patients do not get better and may die, despite taking medicines. This can happen both to patients who join this clinical trial and to patients who do not.

If you decide to join this clinical trial, you will be very closely watched by both your healthcare team and by trial staff to see if you get pneumonia. If you do get pneumonia and take one of the antibiotics used in the trial, you will continue to be very closely watched. Your health is the top priority of your healthcare team and trial staff. They will pay close attention to how you respond to the antibiotic you received as part of the trial. If your healthcare team and trial staff feel the antibiotic is not working for you after [add period of time], you will be given a different antibiotic to try to treat your pneumonia. These will be the regular antibiotics your healthcare team normally uses at this hospital to treat pneumonia.

If you decide NOT to join this clinical trial, you will be watched closely by your healthcare team to see if you get pneumonia. If you do get pneumonia, you will be given the regular antibiotics that your healthcare team normally uses at this hospital to treat pneumonia. Your healthcare team will watch you closely and monitor how you are responding to any treatment. Your health is the top priority of your healthcare team.

If you decide to join the trial or not join the trial, you will receive immediate treatment if you develop pneumonia.

B. Text on how to explain rationale for advance consent and the early enrollment strategy.

1. Original text developed using qualitative interview findings. Presented during online survey #1.

We are trying a new way of enrolling patients into clinical trials for treating pneumonia. This new way is to enroll patients into the trial before they get pneumonia. Patients who will be enrolled are in the intensive care unit and are at high risk for getting pneumonia while in the hospital. We want to find out if enrolling patients early like this can lead to more patients taking part in clinical trials and can speed up the process of developing new medicines for patients in the future.

You do not have pneumonia now. We are talking to you because you have a greater chance of getting pneumonia compared to other patients who are not in the intensive care unit. This means that there is a chance that you could get pneumonia while you are in the hospital. The reasons you might get pneumonia are because you may have been in the hospital for longer than 48 hours or are on a machine that helps you to breathe. There may also be other reasons that put you at a greater chance of getting pneumonia. Your doctor can tell you those reasons.

© 2020 Corneli A et al. *JAMA Network Open.*

If you choose to join this study, you will be agreeing to two parts. First, you agree that study staff can access your hospital records while you are in the hospital. This is so they can see if you get pneumonia during this time. Second, if you do get pneumonia, you agree to receive one of the two study antibiotics that are being tested as part of the trial. What is new about this approach is that you are being asked to consent for these two parts while you are only at risk for getting pneumonia, meaning there is a chance you could get pneumonia. In most trials, you would be asked to join the study only after you had already gotten pneumonia.

We want to make sure that you understand the clinical trial and make your own decision about taking part. If you are unable to make a decision on your own at this time, we want to make sure your legally authorized representative (LAR), if you have one, understands the clinical trial so she/he can make a decision on your behalf. By telling you (and/or your LAR) about the clinical trial now, you have more time to decide if you want to take part. Also, if you get pneumonia later and are asked to think about taking part in the trial at that time, it may be harder to make a decision because you may feel very sick.

If you decide to not join the trial and you get pneumonia, you will be given the standard antibiotic that the hospital uses to treat pneumonia. This might make you unable to take part in the clinical trial because the Food and Drug Administration (FDA) has a rule that patients cannot join pneumonia studies if they have been treated with an antibiotic for more than 24 hours. If you decide now that you do want to take part in the clinical trial, then you will be given one of the two study antibiotics if you do get pneumonia.

It is also possible that you will NOT get pneumonia. If you agree to take part in this clinical trial and do not get pneumonia, you will not need treatment for pneumonia. You will not get either of the study antibiotics.

2. Revised text based on online survey #1 findings. Presented during online survey #2.

We are testing a new way to enroll patients into clinical trials for treating pneumonia. This new way enrolls patients into the trial before they get pneumonia. Only patients who are in the intensive care unit and are at high risk for getting pneumonia while in the hospital can enroll. We want to find out if enrolling patients early leads to more patients taking part in clinical trials on the treatment of pneumonia. This may speed up the process of developing better treatments for pneumonia for patients in the future.

You do not have pneumonia now, and you may never get it. But, you have a greater chance of getting pneumonia during your hospital stay compared to other patients. Research has shown that patients have a greater chance of developing pneumonia if they are in the hospital for longer than 48 hours or if they are on a machine that helps them breathe. There are also other reasons that increase patients' chances of getting pneumonia. Your healthcare team can tell you the specific factors that may apply to you.

© 2020 Corneli A et al. *JAMA Network Open.*

If you choose to join this study, you are agreeing to two parts:

1. You agree that trial staff can access your hospital records while you are in the hospital. This is so they can see if you get pneumonia during this time.
2. If you DO get pneumonia, you agree to receive one of the two antibiotics that are being used in the trial.

What is new about this approach is that you are being asked to consent for both parts of the trial when you do not have pneumonia. In other kinds of clinical trials on pneumonia, patients are asked to join the trial only after they get pneumonia.

We want to make sure that you understand the clinical trial so you can make your own decision about taking part. By telling you about this clinical trial now, you have more time to decide if you want to take part. You can talk with your personal doctor and family about the trial if you wish. Also, it may be easier to make a decision now compared to later if you get pneumonia, because you may feel very sick then.

If you agree to join this clinical trial and you do not get pneumonia, you will not need treatment for pneumonia. We will continue to collect information about you until you leave the intensive care unit (ICU).

If you decide NOT to join the trial and you get pneumonia, you will be given standard antibiotics that the hospital uses to treat pneumonia.

3. Final proposed text.

Reasons for an early enrollment strategy

We are testing a new way to enroll patients into clinical trials for treating pneumonia. This new way enrolls patients into the trial **before** they get pneumonia. Only patients who are in the intensive care unit and are at high risk for getting pneumonia while in the hospital can enroll. We want to find out if enrolling patients early leads to more patients taking part in clinical trials on the treatment of pneumonia. This may improve the process of developing better treatments for pneumonia for patients in the future.

You do not have pneumonia now, and you may never get it. But, you have a greater chance of getting pneumonia during your hospital stay compared to other patients. Research has shown that patients have a greater chance of developing pneumonia if they are in the hospital for longer than 48 hours or if they are on a machine that helps them breathe. There are also other reasons that increase patients' chances of getting pneumonia. Your healthcare team can tell you the specific factors that may apply to you.

If you choose to join this study, you are agreeing to two parts:

© 2020 Corneli A et al. *JAMA Network Open.*

1. You agree that trial staff can access your hospital records while you are in the hospital. This is so they can see if you get pneumonia during this time.
2. If you DO get pneumonia, you agree to receive one of the two antibiotics that are being used in the trial.

What is new about this approach is that you are being asked to consent for both parts of the trial when you do not have pneumonia. In other kinds of clinical trials on pneumonia, patients are asked to join the trial only *after* they get pneumonia.

We want to make sure that you understand the clinical trial so you can make your own decision about taking part. By telling you about this clinical trial now, you have more time to decide if you want to take part. You can talk with your personal doctor and family about the trial if you wish. Also, it may be easier to make a decision now compared to later if you get pneumonia, because you may feel very sick then.

If you agree to join this clinical trial and you do not get pneumonia, you will not need treatment for pneumonia. We will continue to collect information about you until you leave the intensive care unit (ICU).

If you decide NOT to join the trial and you get pneumonia, you will be given the regular antibiotics that the hospital normally uses to treat pneumonia.

C. Text on how to explain noninferiority.

1. Original text developed using qualitative interview findings. Presented during online survey #1.

Note: We chose to make the language about the study drugs applicable for any new drug application rather than using the drug names in our study example. Thus, specific information about imipenem and meropenem and their routine use was not included.

What is the reason for this clinical trial?

We want to find new drugs that treat the types of pneumonia that people get when in the hospital. This is because patients need more medication options. Some patients cannot take certain drugs. Also, some drugs might not work as well as other drugs but have benefits that patients want. For example, some drugs work very well in treating pneumonia but have severe side effects. Patients who want to limit side effects may want another drug that has fewer side effects, even if it may treat pneumonia less well. To be able to have more treatment options, clinical trials must be done to find out if new drugs can treat pneumonia and are safe. When more than one drug is available, patients can choose the trade-offs that work best for them.

<u>What is the purpose of this clinical trial?</u>

The purpose of this clinical trial is to find out if a new drug can treat the types of pneumonia that people get in the hospital. To do this, researchers must test how well the new drug works in treating pneumonia, compared to an existing drug that has already been shown to treat this type of pneumonia. Researchers believe that the new drug can treat pneumonia, but it is possible that it may work less well than the existing drug. Before this trial started, researchers determined how much less well the new drug can work compared to the existing drug and still be acceptable. Researchers believe the difference between the two drugs will likely not impact patients' overall health.

The existing drug is called [insert name]. It has been approved by the U.S. Food and Drug Administration (FDA) for the treatment of pneumonia. The new drug is called [insert name]. It is considered "new" because it has not been approved by the FDA for the treatment of pneumonia. However, it has been approved by the FDA for the treatment of [insert condition(s)]. This means that researchers know some information about how this drug affects people. For example, researchers know some of side effects to expect when this drug is used. The reason researchers feel the new drug is worth testing is because it may offer benefits to patients that the existing drug does not. The best way to find out if the new drug works in treating pneumonia and has any benefits compared to the existing drug is to do the clinical trial.

For patients in this clinical trial who get pneumonia, some will get the new drug by chance and some will get the existing drug by chance. At the end of the trial, the results between the two groups of patients will be compared. If the new drug works less well than the existing drug in treating pneumonia, by more than the amount specified before the trial, researchers will conclude that the new drug does not work in treating pneumonia.

2. Revised text based on online survey #1 findings. Presented during online survey #2.

<u>What is the reason for this kind of clinical trial?</u>

We want to identify antibiotics that can treat the types of pneumonia that people get when in the hospital. This is because patients need more medication options than are available today. Some patients cannot take certain antibiotics. Also, some antibiotics might not work as well as other antibiotics, but they have benefits that patients want, like fewer side effects. For example, one antibiotic may work very well in treating pneumonia but has severe side effects. Another antibiotic may not treat pneumonia as well but has fewer side effects. Some patients and doctors may prefer the antibiotic that has fewer side effects, even if it may not treat pneumonia as well. When more than one antibiotic is available, patients and their doctors can consider the trade-offs and choose the antibiotic that is best for them.

One way to identify more antibiotics for pneumonia is to find out if existing antibiotics that were approved for another condition can also treat pneumonia. Often drugs that were approved for treating one condition can be found to work in treating another condition. However, clinical trials

must be done first to find out if certain existing antibiotics approved for other conditions can also treat pneumonia and are considered safe.

What is the purpose of this clinical trial?
One purpose of this clinical trial is to find out if an existing antibiotic called [insert existing antibiotic name] can treat the type of pneumonia that people get in the hospital. The existing antibiotic has not been approved by the Food and Drug Administration (FDA) for the treatment of pneumonia. However, it has been approved by the FDA for the treatment of [insert condition(s)]. This means that researchers already know some information about how this antibiotic affects people. For example, researchers know some of the side effects to expect when this antibiotic is used.

To find out if [insert existing antibiotic name], the existing antibiotic, can also treat pneumonia, researchers must test how well it works in treating pneumonia when compared to an antibiotic that has already been shown to treat pneumonia that people get in the hospital and was approved by the FDA. The approved antibiotic is called [insert approved antibiotic name].

Researchers believe that [insert existing antibiotic name], the existing drug, can treat pneumonia, but it may not work as well as [insert approved antibiotic name], the antibiotic that has already been approved for treating pneumonia. The reason researchers feel [insert existing antibiotic name] is worth testing is because it may offer benefits to patients with pneumonia that [insert approved antibiotic name] does not. These possible benefits include [list benefits here]. Another purpose of the clinical trial is to find out if [insert existing antibiotic name] has any benefits compared to [insert approved antibiotic name].

Patients who join this clinical trial and who get pneumonia will be placed in one of two groups:

- Patients in group one will get [insert existing antibiotic name], the existing drug that has been approved for [insert condition(s)].
- Patients in group two will get [insert approved antibiotic name], the antibiotic that has already been approved for treating pneumonia.

A process called “randomization,” which is like flipping a coin, is used to find out which group participants will be in. This is done because it is not known which treatment works better for pneumonia.

At the end of the trial, the results between the two groups of patients will be compared. If [insert existing antibiotic name], the existing drug, does not work as well as [insert approved antibiotic name], the approved drug, in treating pneumonia—or works less well than expected—then the researchers will conclude that [insert existing antibiotic name] does not work well enough to give to patients to treat pneumonia.

© 2020 Corneli A et al. *JAMA Network Open.*

3. Final proposed text.

What is the reason for this kind of clinical trial?
We want to identify antibiotics that can treat the types of pneumonia that people get when in the hospital. This is because patients need more medication options than are available today. Some patients cannot take certain antibiotics. Also, some antibiotics might not work as well as other antibiotics, but they have benefits that patients want, like fewer side effects. For example, one antibiotic may work very well in treating pneumonia but has severe side effects. Another antibiotic may not treat pneumonia as well but has fewer side effects. Some patients and doctors may prefer the antibiotic that has fewer side effects, even if it may not treat pneumonia as well. When more than one antibiotic is available, patients and their doctors can consider the trade-offs and choose the antibiotic that is best for them.

One way to identify more antibiotics for pneumonia is to find out if existing antibiotics that were approved for another condition can also treat pneumonia. However, clinical trials must be done first to find out if certain existing antibiotics approved for other conditions can also treat pneumonia and are considered safe.

What is the purpose of this clinical trial?
One purpose of this clinical trial is to find out if an existing antibiotic called [insert existing antibiotic name] can treat the type of pneumonia that people get in the hospital. The existing antibiotic has not been approved by the Food and Drug Administration (FDA) for the treatment of pneumonia. However, it has been approved by the FDA for the treatment of [insert condition(s)]. This means that researchers already know some information about how this antibiotic affects people. For example, researchers know some of the side effects to expect when this antibiotic is used.

To find out if [insert existing antibiotic name], the existing antibiotic, can also treat pneumonia, researchers must test how well it works in treating pneumonia when compared to an antibiotic that has already been shown to treat pneumonia that people get in the hospital. The approved antibiotic is called [insert approved antibiotic name].

Researchers believe that [insert existing antibiotic name], the existing drug, can treat pneumonia, but it may not work as well as [insert approved antibiotic name], the antibiotic that has already been approved for treating pneumonia. The reason researchers feel [insert existing antibiotic name] is worth testing is because it may offer benefits to patients with pneumonia that [insert approved antibiotic name] does not. These possible benefits include [list benefits here].

Patients who join this clinical trial and who get pneumonia will be placed in one of two groups:

- Patients in group one will get [insert existing antibiotic name], the existing drug that has been approved for [insert condition(s)].
- Patients in group two will get [insert approved antibiotic name], the antibiotic that has already been approved for treating pneumonia.

© 2020 Corneli A et al. *JAMA Network Open.*

A process called "randomization," which is like flipping a coin, is used to decide which group participants will be in. This is done because it is not known which treatment works better for pneumonia.

At the end of the trial, the results between the two groups of patients will be compared. If [insert existing antibiotic name], the existing drug, does not work as well as [insert approved antibiotic name], the approved drug, in treating pneumonia—or works less well than expected—then the researchers will conclude that [insert existing antibiotic name] does not work well enough to give to patients to treat pneumonia.

© 2020 Corneli A et al. *JAMA Network Open.*

eAppendix 4: Responses to Online Survey #2

This eAppendix provides the frequency data for each statement or group of statements assessed by stakeholders during the second online survey.

Stakeholders could choose one of the following two responses for each statement or groups of statements: (1) “Important: Should remain” and (2) “Unimportant: Remove”. Stakeholders were asked to provide their rationale in an open-ended field if they selected “Unimportant: Remove”. They could also provide overall comments on the statement or group of statements.

For the response, “Unimportant: Remove”:

1. We recoded the stakeholder responses and placed them in their own category when, in the open-ended field, stakeholders:

    a. Suggested text modifications to the existing statement(s)
    b. Asked questions or provided comments about the study that were unrelated to removing the statement or group of statements

2. We kept responses as reported (i.e., did not re-code) when respondents suggested to remove one of the statements in a multi-statement section.

All re-coding was verified by two analysts.

© 2020 Corneli A et al. *JAMA Network Open.*

Category #1: Rationale for advance consent and early enrollment

A. **Number of stakeholder respondents and response rate**

Stakeholder Group	n (%)
Patients	14 (100)
LARs	9 (90)
Coordinators	4 (100)
Investigators	5 (100)
IRB	8 (100)
Total completion	40 (98)

B. **Statements or groups of statements**

1. **Statement: We are testing a new way to enroll patients into clinical trials for treating pneumonia. This new way enrolls patients into the trial before they get pneumonia. Only patients who are in the intensive care unit and are at high risk for getting pneumonia while in the hospital can enroll.**

	Stakeholder Group (n)					
Response Option	Patients	LARs	Coordinators	Investigators	IRB	Total
Important: Should remain	14	9	4	5	8	40
Unimportant: Remove	0	0	0	0	0	0

2. **Statement: We want to find out if enrolling patients early leads to more patients taking part in clinical trials on the treatment of pneumonia. This may speed up the process of developing better treatments for pneumonia for patients in the future.**

	Stakeholder Group (n)					
Response Option	Patients	LARs	Coordinators	Investigators	IRB	Total
Important: Should remain	13	8	4	5	8	38
Unimportant: Remove	1	1	0	0	0	2

3. **Statement: You do not have pneumonia now, and you may never get it.**

	Stakeholder Group (n)					
Response Option	Patients	LARs	Coordinators	Investigators	IRB	Total
Important: Should remain	13	8	4	5	8	38
Unimportant: Remove	1	1	0	0	0	2

4. **Statement: But, you have a greater chance of getting pneumonia during your hospital stay compared to other patients.**

	Stakeholder Group (n)					
Response Option	Patients	LARs	Coordinators	Investigators	IRB	Total
Important: Should remain	13	6	4	5	8	36
Unimportant: Remove	1	3	0	0	0	4

© 2020 Corneli A et al. *JAMA Network Open.*

5. **Statement: Research has shown that patients have a greater chance of developing pneumonia if they are in the hospital for longer than 48 hours or if they are on a machine that helps them breathe.**

Response Option	Stakeholder Group (n)					Total
	Patients	LARs	Coordinators	Investigators	IRB	
Important: Should remain	14	9	4	5	7	39
Unimportant: Remove	0	0	0	0	1	1

6. **Statement: There are also other reasons that increase patients' chances of getting pneumonia. Your healthcare team can tell you the specific factors that may apply to you.**

Response Option	Stakeholder Group (n)					Total
	Patients	LARs	Coordinators	Investigators	IRB	
Important: Should remain	14	7	3	3	7	34
Unimportant: Remove	0	2	1	0	1	4
Recoded from "Unimportant: Remove"	0	0	0	2	0	2

7. **Statement: If you choose to join this study, you are agreeing to two parts: (1) You agree that trial staff can access your hospital records while you are in the hospital. This is so they can see if you get pneumonia during this time. (2) If you DO get pneumonia, you agree to receive one of the two antibiotics that are being used in the trial.**

Response Option	Stakeholder Group (n)					Total
	Patients	LARs	Coordinators	Investigators	IRB	
Important: Should remain	14	9	4	5	8	40
Unimportant: Remove	0	0	0	0	0	0

8. **Statement: What is new about this approach is that you are being asked to consent for both parts of the trial when you do not have pneumonia. In other kinds of clinical trials on pneumonia, patients are asked to join the trial only after they get pneumonia.**

Response Option	Stakeholder Group (n)					Total
	Patients	LARs	Coordinators	Investigators	IRB	
Important: Should remain	12	9	4	4	8	37
Unimportant: Remove	1	0	0	1	0	2
Recoded from "Unimportant: Remove"	1	0	0	0	0	1

© 2020 Corneli A et al. *JAMA Network Open.*

9. Statement: We want to make sure that you understand the clinical trial so you can make your own decision about taking part. By telling you about this clinical trial now, you have more time to decide if you want to take part. You can talk with your personal doctor and family about the trial if you wish. Also, it may be easier to make a decision now compared to later if you get pneumonia, because you may feel very sick then.

Response Option	Stakeholder Group (n)					Total
	Patients	LARs	Coordinators	Investigators	IRB	
Important: Should remain	13	9	4	4	8	38
Unimportant: Remove	1	0	0	1	0	2

10. Statement: If you agree to join this clinical trial and you do <u>not</u> get pneumonia, you will not need treatment for pneumonia. We will continue to collect information about you until you leave the intensive care unit (ICU).

Response Option	Stakeholder Group (n)					Total*
	Patients	LARs	Coordinators	Investigators	IRB	
Important: Should remain	12	9	4	5	8	38
Unimportant: Remove	1	0	0	0	0	1

*A response from a patient is missing.

11. Statement: If you decide NOT to join the trial and you get pneumonia, you will be given standard antibiotics that the hospital uses to treat pneumonia.

Response Option	Stakeholder Group (n)					Total*
	Patients	LARs	Coordinators	Investigators	IRB	
Important: Should remain	14	8	4	5	8	39
Unimportant: Remove	0	0	0	0	0	0

*A response from a LAR is missing.

© 2020 Corneli A et al. *JAMA Network Open.*

Category #2: Reassurances

A. Number of stakeholder respondents and response rate

Stakeholder Group	n (%)
Patients	14 (100)
LARs	9 (90)
Coordinators	4 (100)
Investigators	5 (100)
IRB	8 (100)
Total completion	40 (98)

B. Statements or groups of statements

1. Statement: People who get pneumonia when in the hospital are very sick. Some of these patients do not get better and may die, despite taking medicines. This can happen to patients who join this clinical trial and to patients who do not.

	Stakeholder Group (n)					
**Response Option**	**Patients**	**LARs**	**Coordinators**	**Investigators**	**IRB**	**Total**
Important: Should remain	13	9	4	5	7	38
Unimportant: Remove	1	0	0	0	1	2

2. Statement: If you decide to join this clinical trial, you will be very closely watched by both your healthcare team and by trial staff to see if you get pneumonia.

	Stakeholder Group (n)					
**Response Option**	**Patients**	**LARs**	**Coordinators**	**Investigators**	**IRB**	**Total***
Important: Should remain	14	7	4	5	8	38
Unimportant: Remove	0	0	0	0	0	0
Recoded from “Unimportant: Remove”	0	1	0	0	0	1

*A response from a LAR is missing.

3. Statement: If you do get pneumonia and take one of the antibiotics used in the trial, you will continue to be very closely watched.

	Stakeholder Group (n)					
**Response Option**	**Patients**	**LARs**	**Coordinators**	**Investigators**	**IRB**	**Total**
Important: Should remain	13	8	4	5	8	38
Unimportant: Remove	1	0	0	0	0	1
Recoded from “Unimportant: Remove”	0	1	0	0	0	1

© 2020 Corneli A et al. *JAMA Network Open.*

4. **Statement: Your health is the top priority of your healthcare team and trial staff. They will pay close attention to how you respond to the antibiotic you received as part of the trial.**

**Response Option**	**Stakeholder Group (n)**					**Total**
	**Patients**	**LARs**	**Coordinators**	**Investigators**	**IRB**	
Important: Should remain	14	9	4	5	7	39
Unimportant: Remove	0	0	0	0	0	0

*A response from an IRB representative is missing.

5. **Statement: If your healthcare team and trial staff feel the antibiotic is not working for you after [add period of time], you will be given a different antibiotic to try to treat your pneumonia. These will be the standard antibiotics your healthcare team normally uses at this hospital to treat pneumonia.**

**Response Option**	**Stakeholder Group (n)**					**Total**
	**Patients**	**LARs**	**Coordinators**	**Investigators**	**IRB**	
Important: Should remain	14	9	4	5	8	40
Unimportant: Remove	0	0	0	0	0	0

6. **Statement: If you decide to NOT join this clinical trial, you will be watched closely by your healthcare team to see if you get pneumonia.**

**Response Option**	**Stakeholder Group (n)**					**Total**
	**Patients**	**LARs**	**Coordinators**	**Investigators**	**IRB**	
Important: Should remain	13	8	4	5	8	38
Unimportant: Remove	1	0	0	0	0	1
Recoded from “Unimportant: Remove”	0	1	0	0	0	1

7. **Statement: If you do get pneumonia, you will be given the standard antibiotics that your healthcare team normally uses at this hospital to treat pneumonia.**

**Response Option**	**Stakeholder Group (n)**					**Total**
	**Patients**	**LARs**	**Coordinators**	**Investigators**	**IRB**	
Important: Should remain	14	8	4	5	7	38
Unimportant: Remove	0	1	0	0	0	1
Recoded from “Unimportant: Remove”	0	0	0	0	1	1

8. **Statement: Your healthcare team will watch you closely and monitor how you are responding to any treatment. Your health is the top priority of your healthcare team.**

**Response Option**	**Stakeholder Group (n)**					**Total**
	**Patients**	**LARs**	**Coordinators**	**Investigators**	**IRB**	
Important: Should remain	14	8	4	5	7	38
Unimportant: Remove	0	0	0	0	1	1
Recoded from “Unimportant: Remove”	0	1	0	0	0	1

© 2020 Corneli A et al. *JAMA Network Open.*

Category #3: Noninferiority

A. Number of stakeholder respondents and response rate

Stakeholder Group	n (%)
Patients	14 (100)
LARs	9 (90)
Coordinators	4 (100)
Investigators	5 (100)
IRB	8 (100)
Total completion	40 (98)

B. Statements or groups of statements

1. Statement: What is the reason for this kind of clinical trial? We want to identify antibiotics that can treat the types of pneumonia that people get when in the hospital. This is because patients need more medication options than are available today.

Response Option	Stakeholder Group (n)					Total
	Patients	LARs	Coordinators	Investigators	IRB	
Important: Should remain	14	9	4	5	8	40
Unimportant: Remove	0	0	0	0	0	0

2. Statement: Some patients cannot take certain antibiotics. Also, some antibiotics might not work as well as other antibiotics, but they have benefits that patients want, like fewer side effects.

Response Option	Stakeholder Group (n)					Total*
	Patients	LARs	Coordinators	Investigators	IRB	
Important: Should remain	14	8	4	4	7	37
Unimportant: Remove	0	0	0	1	1	2

*A response from a LAR is missing.

3. Statement: For example, one antibiotic may work very well in treating pneumonia but has severe side effects. Another antibiotic may not treat pneumonia as well but has fewer side effects.

Response Option	Stakeholder Group (n)					Total*
	Patients	LARs	Coordinators	Investigators	IRB	
Important: Should remain	14	8	4	3	4	33
Unimportant: Remove	0	0	0	2	2	4
Recoded from "Unimportant: Remove"	0	0	0	0	1	1

*A response from a LAR and an IRB representative is missing.

© 2020 Corneli A et al. *JAMA Network Open.*

4. **Statement: Some patients and doctors may prefer the antibiotic that has fewer side effects, even if it may not treat pneumonia as well.**

Response Option	Stakeholder Group (n)					Total
	Patients	LARs	Coordinators	Investigators	IRB	
Important: Should remain	13	6	4	3	6	32
Unimportant: Remove	0	2	0	2	2	6
Recoded from "Unimportant: Remove"	1	1	0	0	0	2

5. **Statement: When more than one antibiotic is available, patients and their doctors can consider the trade-offs and choose the antibiotic that is best for them.**

Response Option	Stakeholder Group (n)					Total*
	Patients	LARs	Coordinators	Investigators	IRB	
Important: Should remain	13	8	4	5	8	38
Unimportant: Remove	0	0	0	0	0	0
Recoded from "Unimportant: Remove"	1	0	0	0	0	1

*A response from a LAR is missing.

6. **Statement: One way to identify more antibiotics for pneumonia is to find out if existing antibiotics that were approved for another condition can also treat pneumonia. Often drugs that were approved for treating one condition can be found to work in treating another condition.**

Response Option	Stakeholder Group (n)					Total*
	Patients	LARs	Coordinators	Investigators	IRB	
Important: Should remain	13	8	4	5	8	38
Unimportant: Remove	1	0	0	0	0	1

***** *A response from a LAR is missing.

7. **Statement: However, clinical trials must be done first to find out if certain existing antibiotics approved for other conditions can also treat pneumonia and are considered safe.**

Q62_3	Stakeholder Group (n)					Total
	Patients	LARs	Coordinators	Investigators	IRB	
Important: Should remain	11	7	4	5	8	35
Unimportant: Remove	1	1	0	0	0	2
Recoded from "Unimportant: Remove"	2	1	0	0	0	3

© 2020 Corneli A et al. *JAMA Network Open.*

8. **Statement: What is the purpose of this clinical trial? One purpose of this clinical trial is to find out if an existing antibiotic called [insert existing antibiotic name] can treat the type of pneumonia that people get in the hospital.**

Response Option	Stakeholder Group (n)					Total
	Patients	LARs	Coordinators	Investigators	IRB	
Important: Should remain	13	9	4	5	8	39
Unimportant: Remove	0	0	0	0	0	0
Recoded from "Unimportant: Remove"	1	0	0	0	0	1

9. **Statement: The existing antibiotic has not been approved by the Food and Drug Administration (FDA) for the treatment of pneumonia. However, it has been approved by the FDA for the treatment of [insert condition(s)].**

Response Option	Stakeholder Group (n)					Total*
	Patients	LARs	Coordinators	Investigators	IRB	
Important: Should remain	13	8	4	5	8	38
Unimportant: Remove	0	0	0	0	0	0
Recoded from "Unimportant: Remove"	1	0	0	0	0	1

*A response from a LAR is missing.

10. **Statement: This means that researchers already know some information about how this antibiotic affects people. For example, researchers know some of the side effects to expect when this antibiotic is used.**

Response Option	Stakeholder Group (n)					Total
	Patients	LARs	Coordinators	Investigators	IRB	
Important: Should remain	13	8	4	4	8	37
Unimportant: Remove	0	1	0	1	0	2
Recoded from "Unimportant: Remove"	1	0	0	0	0	1

11. **Statement: To find out if [insert existing antibiotic name], the existing antibiotic, can also treat pneumonia, researchers must test how well it works in treating pneumonia when compared to an antibiotic that has already been shown to treat pneumonia that people get in the hospital and was approved by the FDA. The approved antibiotic is called [insert approved antibiotic name].**

Response Option	Stakeholder Group (n)					Total*
	Patients	LARs	Coordinators	Investigators	IRB	
Important: Should remain	13	9	4	5	8	39
Unimportant: Remove	0	0	0	0	0	0

*A response from a patient is missing.

© 2020 Corneli A et al. *JAMA Network Open.*

12. **Statement: Researchers believe that [insert existing antibiotic name], the existing drug, can treat pneumonia, but it may not work as well as [insert approved antibiotic name], the antibiotic that has already been approved for treating pneumonia.**

Response Option	Stakeholder Group (n)					Total
	Patients	LARs	Coordinators	Investigators	IRB	
Important: Should remain	13	9	4	5	8	39
Unimportant: Remove	0	0	0	0	0	0
Recoded from “Unimportant: Remove”	1	0	0	0	0	1

13. **Statement: The reason researchers feel [insert existing antibiotic name] is worth testing is because it may offer benefits to patients with pneumonia that [insert approved antibiotic name] does not.**

Response Option	Stakeholder Group (n)					Total*
	Patients	LARs	Coordinators	Investigators	IRB	
Important: Should remain	13	8	4	5	8	38
Unimportant: Remove	1	0	0	0	0	1

*A response from a LAR is missing.

14. **Statement: These possible benefits include [list benefits here].**

Response Option	Stakeholder Group (n)					Total
	Patients	LARs	Coordinators	Investigators	IRB	
Important: Should remain	14	8	4	5	6	37
Unimportant: Remove	0	0	0	0	1	1
Recoded from “Unimportant: Remove”	0	1	0	0	1	2

15. **Statement: Another purpose of the clinical trial is to find out if [insert existing antibiotic name] has any benefits compared to [insert approved antibiotic name].**

Response Option	Stakeholder Group (n)					Total*
	Patients	LARs	Coordinators	Investigators	IRB	
Important: Should remain	12	7	3	3	8	33
Unimportant: Remove	1	1	1	2	0	5
Recoded from “Unimportant: Remove”	1	0	0	0	0	1

*A response from a LAR is missing.

16. **Statement: Patients who join this clinical trial and who get pneumonia will be placed in one of two groups: (1)Patients in group one will get [insert existing antibiotic name], the existing drug that has been approved for [insert condition(s)].(2) Patients in group two will get [insert approved antibiotic name], the antibiotic that has already been approved for treating pneumonia.**

**Response Option**	**Stakeholder Group (n)**					**Total**
	**Patients**	**LARs**	**Coordinators**	**Investigators**	**IRB**	
Important: Should remain	12	9	4	5	8	38
Unimportant: Remove	2	0	0	0	0	2

17. **Statement: A process called "randomization," which is like flipping a coin, is used to find out which group participants will be in.**

**Response Option**	**Stakeholder Group (n)**					**Total**
	**Patients**	**LARs**	**Coordinators**	**Investigators**	**IRB**	
Important: Should remain	14	7	4	5	8	38
Unimportant: Remove	0	2	0	0	0	2

18. **Statement: At the end of the trial, the results between the two groups of patients will be compared.**

**Response Option**	**Stakeholder Group (n)**					**Total***
	**Patients**	**LARs**	**Coordinators**	**Investigators**	**IRB**	
Important: Should remain	14	8	4	4	8	38
Unimportant: Remove	0	0	0	1	0	1

*A response from a LAR is missing.

19. **Statement: If [insert existing antibiotic name], the existing drug, does not work as well as [insert approved antibiotic name], the approved drug, in treating pneumonia - or works less well than expected - then the researchers will conclude that [insert existing antibiotic name] does not work well enough to give to patients to treat pneumonia.**

**Response Option**	**Stakeholder Group (n)**					**Total***
	**Patients**	**LARs**	**Coordinators**	**Investigators**	**IRB**	
Important: Should remain	13	8	3	3	6	33
Unimportant: Remove	1	0	1	2	2	6

*A response from a LAR is missing.

© 2020 Corneli A et al. *JAMA Network Open.*
